# Altered Expression of Genes Associated with Major Neurotransmitter Systems in the Reward-Related Brain Regions of Mice with Positive Fighting Experience

**DOI:** 10.3390/ijms232113644

**Published:** 2022-11-07

**Authors:** Dmitry A. Smagin, Anna G. Galyamina, Irina L. Kovalenko, Natalia N. Kudryavtseva

**Affiliations:** Neuropathology Modeling Laboratory, Neurogenetics of Social Behavior Sector, FRC Institute of Cytology and Genetics SB RAS, 630090 Novosibirsk, Russia

**Keywords:** DEGs, positive fighting experience, addiction-like state, mice

## Abstract

The main neurotransmitters in the brain—dopamine, γ-aminobutyric acid (GABA), glutamate, and opioids—are recognized to be the most important for the regulation of aggression and addiction. The aim of this work was to study differentially expressed genes (DEGs) in the main reward-related brain regions, including the ventral tegmental area (VTA), dorsal striatum (STR), ventral striatum (nucleus accumbens, NAcc), prefrontal cortex (PFC), and midbrain raphe nuclei (MRNs), in male mice with 20-day positive fighting experience in daily agonistic interactions. Expression of opioidergic, catecholaminergic, glutamatergic, and GABAergic genes was analyzed to confirm or refute the influence of repeated positive fighting experience on the development of “addiction-like” signs shown in our previous studies. High-throughput RNA sequencing was performed to identify differentially expressed genes in the brain regions of chronically aggressive mice. In the aggressive mice, upregulation of opioidergic genes was shown (*Oprk1* in VTA, *Pdyn* in NAcc, *Penk* in PFC, and *Oprd1* in MRNs and PFC), as was downregulation of genes *Opcml* and *Oprk1* in STR and *Pomc* in VTA and NAcc. Upregulation of catecholaminergic genes in VTA (*Ddc* and *Slc6a2*) and in NAcc (*Th* and *Drd2*) and downregulation of some differentially expressed genes in MRNs (*Th*, *Ddc*, *Dbh*, *Drd2*, *Slc18a2*, and *Sncg*) and in VTA (*Adra2c*, *Sncg*, and *Sncb*) were also documented. The expression of GABAergic and glutamatergic genes that participate in drug addiction changed in all brain regions. According to literature data, the proteins encoded by genes *Drd2*, *Oprk1*, *Oprd1*, *Pdyn*, *Penk*, and *Pomc* are directly involved in drug addiction in humans. Thus, our results confirm our earlier claim about the formation of addiction-like signs following repeated positive fighting experience in mice, as shown previously in our biobehavioral studies.

## 1. Introduction

The main neurotransmitters—dopamine (DA), serotonin (5-HT), γ-aminobutyric acid (GABA), and glutamate—are recognized as the most important neurotransmitters for the mechanisms of aggression [1,2,3,4,5,6,7]. A role of endogenous opioids has been shown in offensive aggression [8]. Aggression is also closely associated with activation or inhibition of opioidergic receptors and is strongly influenced by drugs [1,9]. 5-HT is also considered an active player in the inhibition of control over aggression [10,11,12,13,14,15,16]. It is well known that all these neurotransmitter systems are involved in the development of reward and addiction.

In our early study, we found the activation of brain dopaminergic systems in male mice winning in daily agonistic interactions with unfamiliar conspecifics [17]. This activation was detected as elevated DOPAC (3,4-dihydroxyphenylacetic acid) levels or/and increased DOPAC/DA ratios in the amygdala, nucleus accumbens, and dorsal striatum in the winners as compared with controls.

Pharmacological data have also confirmed changes of dopaminergic activity during positive fighting experience. Haloperidol (an antagonist of dopaminergic receptors [18]) and D1 receptor antagonist SCH-23390 [19] effectively decreased aggression in male mice with 2-day experience of aggression and were ineffective in the same doses in mice with 20-day winning experience. These observations indicate the desensitization of DA receptors induced by chronic activation of dopaminergic systems after experience of repeated aggression. Experiments have also revealed development of tolerance to an opioid receptor antagonist (naltrexone) [20,21,22]: blockade of opioid receptors had no significant effect on the level of aggression in winners with 20-day positive fighting experience in contrast to 2-day winners. Activation of the opioidergic system induced by an agonist of mu-opioid receptors (morphine) [23] and by a kappa-opioid receptor agonist (U-50,488H) revealed desensitization of opioid receptors: in some experiments, the winners hardly responded to treatment with these drugs in comparison with a control, which responded to the drug injections by behavioral changes [24]. 

Using reverse transcription (RT) and then qPCR, we also found that 20-day repeated aggression is accompanied by upregulation of the *Th* gene encoding tyrosine hydroxylase, the *Slc6a3 (Dat1)* gene encoding DA transporter, and the *Snca* gene encoding α-synuclein [25,26] in the ventral tegmental area (VTA) of winners and downregulation of *Comt* in the midbrain [27]. Moreover, the expression of genes *Th* and *Slc6a3* stays enhanced in VTA for at least 2 weeks after discontinuation of agonistic interactions and correlates with the level of aggressiveness of winners [26].

It has been suggested that positive fighting experience, like other basic behaviors, provides a permanent reward to the winners, hence a tendency to repeat acts of aggression. Aggressive motivation becomes generalized and dominant in any social situation. Chronically aggressive male mice, as a rule demonstrating unmotivated aggression, attack any conspecifics in any situations [28,29]. There is evidence that the winners demonstrate impaired communication and sociability [30] and prolonged persistence of changes in behaviors and emotional states after 2 weeks without agonistic interactions. Generally, our behavioral and pharmacological data have allowed us to hypothesize the development of an addiction-like state in the winners as a result of positive fighting experience in daily agonistic interactions [29]. Increased aggressiveness after a period of fighting deprivation [31] confirms the development of psychopathology in male mice following repeated aggression. Additionally, other authors have noticed signs of pathological aggression [5,32,33] and signs of “addiction-like” alterations in aggressive mice [34,35].

The aim of the present work was to analyze differential expression of well-known genes encoding proteins that are involved in the development of reward, drug abuse, addiction, and aggression [2,3,4,6,8,36,37]. Primarily, these are the opioidergic and catecholaminergic (CAergic) genes, and their mRNA expression was analyzed to confirm or refute the development of an addiction-like state under the influence of repeated positive fighting experience, as shown in our previous studies [29]. Furthermore, we took into consideration data showing that abnormalities in glutamatergic [38] and/or GABAergic neurotransmission may underlie resting-state functional deficits in drug addiction [39] and aggression [6]. 

Transcriptomic analysis was performed in the main reward-related brain regions that are involved in the mechanisms of positive reinforcement [40,41,42]: these are the VTA, dorsal striatum (STR), ventral striatum (NAcc), prefrontal cortex (PFC), and midbrain raphe nuclei (MRNs) in male mice with 20-day positive fighting experience in daily agonistic interactions. Only the mice that demonstrated permanent strongest, uncontrollable maniacal excessive aggression during a 20-day period were analyzed. The study is carried out within the framework of the direction we are developing “Functional neurogenomics of pathological conditions” with use of the sensory contact model [43], renamed later as the chronic social conflicts model, allows to obtain male mice with an aggressive type of behavior [44]. 

## 2. Results

### 2.1. Agonistic Behavior

Among male mice with daily experience of aggression for 20 tests (days), highly aggressive animals were identified (Table 1), which in daily agonistic interactions demonstrated a high level of aggressiveness, stopping during an attack only for a short rest, after which they again attacked the loser, thus demonstrating unmotivated aggression and attacking another male, regardless of its behavior (active, passive, or full defense). Other aggressive mice usually stopped attacking if the loser showed postures of complete submission or immobility. Moreover, changes in social behaviors and psychoemotional states after long positive fighting experience that never appeared in control animals were noted in the winners and included the demonstration of abnormal aggression, strong hostility, an anxious state, uncontrollable behavior, disturbances in social recognition, hyperactivity, and stereotypic and hyperkinetic reactions [28,29,44]. In any social situation, these aggressive mice demonstrated high motivation to attack, bite, and chase any partners. 

The total time of attacking behavior, the average time of one attack, and the total time of hostile behavior were significantly longer in highly aggressive mice (which were subjected to transcriptome analysis) than in less aggressive mice (Table 1). Behavioral observations showed that 50% of highly aggressive mice additionally displayed total extended time of unmotivated self-grooming behavior (61.3 ± 13.8 s), which may be regarded as stereotypical behavior. Of note, the number of jerks (winces) was significantly higher in the aggressive mice compared with highly aggressive mice (15.9 vs. 8.5 s), possibly as a result of ambivalence when a decision (to attack or not) is making in situation of agonistic interactions. Aggressive mice, as a rule, first sniffed the partner and only then attacked it. 

### 2.2. Neurotranscriptomic Data

All DEGS in different brain regions are presented in Supplementary Table S1. 

#### 2.2.1. DEGs in the VTA of Highly Aggressive Mice 

**Figure 1:**
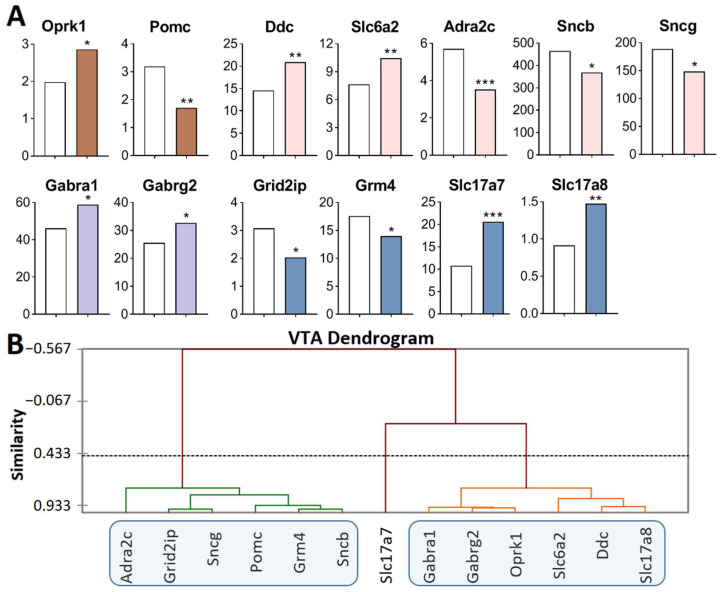
DEGs in the VTA of highly aggressive mice. (**A**) FPKM of the DEGs in the VTA (Supplementary Table S1). White bars—controls; colored bars—winners: brown—opioidergic genes; pink—CAergic genes; lilac—GABAergic genes; violet—glutamatergic genes. * *p* < 0.05; ** *p* < 0.01; *** *p* < 0.001. (**B**) AHC based on 13 reference DEGs’ expression profiles (additional information: Supplementary Table S2). Similarity: Pearson’s correlation coefficient. Agglomeration method: unweighted pair-group average. The main clusters are highlighted with a blue rounded rectangle.

Analysis of the RNA-Seq database of the VTA [45,46] revealed DEGs among genes of interest that participate in the rewarding processes (Figure 1A; Supplementary Table S1). 

Opioidergic genes: *Oprk1* was upregulated (*p* = 0.01) and *Pomc* was downregulated (*p* = 0.002); CAergic genes: *Ddc* (*p* = 0.003) and *Slc6a2* (*p* = 0.007) were upregulated; *Adra2c* (*p* < 0.001), *Sncb* (*p* = 0.045), and *Sncg* (*p* = 0.03) were downregulated; GABAergic genes: *Gabra1* and *Gabrg2* were upregulated (*p* = 0.035 and 0.025, respectively); Glutamatergic genes: *Slc17a7* (*p* < 0.001, q = 0.02) and *Slc17a8* (*p* = 0.005) were upregulated, and *Grm4* (*p* = 0.039) and *Grid2ip* (*p* = 0.013) were downregulated.

Three gene clusters were identified showing relationships among DEGs after repeated aggression (Figure 1B; Supplementary Table S2). The first cluster contains genes whose expression diminished: *Pomc,* proopiomelanocortin; CAergic genes *Adra2c, Sncb,* and *Sncg*; and glutamatergic genes *Grm4* and *Grid2ip* (encoding metabotropic and ionotropic receptors). The expression and number of correlations with other genes (Supplementary Table S3) were the highest for the *Sncg* gene, which encodes a member of the synuclein family of proteins, which are believed to take part in the pathogenesis of neurodegenerative diseases together with *Sncb* (GeneCards). It is likely that it is the downregulation of this gene that leads to a decrease in the expression of the genes in this cluster. 

The second cluster of genes contains the upregulated opioidergic *Oprk1* gene encoding kappa-opioid receptors, CAergic genes *Ddc* and *Slc6a2*, glutamatergic gene *Slc17a8*, and genes *Gabra1* and *Gabrg2* coding for GABA receptors (Figure 1B, Supplementary Table S2). The expression and number of correlations with other genes (Supplementary Table S3) were the highest for genes *Gabrg2* and *Oprk1*, which correlated with each other too (R = 0.957).

It can be assumed that the glutamatergic system is activated, as evidenced by high expression of genes of vesicular glutamate transporters *Slc17a7* and *Slc17a8*, against the background of downregulation of the metabotropic glutamate *Grm4* gene and *Grid2ip* gene encoding glutamate receptors. In this context, the expression of the gene encoding adrenergic receptor *Adra2c* declined. The lowest number of correlations was found for *Slc17a7*, *Pomc*, *Slc6a2*, and *Adra2c* (Supplementary Table S3). 

Thus, the analysis of the DEGs of interest (Figure 1A) together with experimental data published by us earlier [17,25] indicates activation of CAergic systems that are accompanied by upregulation of genes encoding the main enzyme for the synthesis (*Th* gene), a gene coding for DA-DOPA-decarboxylase of aromatic amino acids (*Ddc* gene), and genes encoding noradrenaline and DA transporters (*Slc6a2* and *Slc6a3*, respectively). 

#### 2.2.2. DEGs in the NAcc of Highly Aggressive Mice

**Figure 2:**
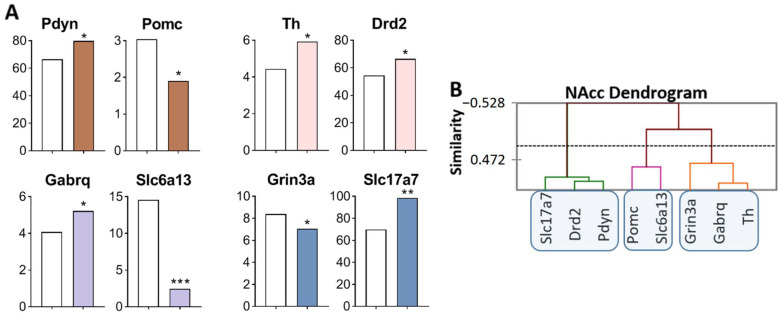
DEGs in the NAcc of highly aggressive mice. (**A**) FPKM of the DEGs in the NAcc (Supplementary Table S1). White bars: controls; colored bars—winners: brown—opioidergic genes; pink - CAergic genes; lilac—GABAergic genes; violet—glutamatergic genes. * *p* < 0.05; ** *p* < 0.01; *** *p* < 0.001. (**B**) AHC based on expression profiles of eight reference DEGs (additional information: Supplementary Table S2). Similarity: Pearson’s correlation coefficient. Agglomeration method: Unweighted pair-group average. The main clusters are highlighted with a blue rounded rectangle.

It has been shown (Figure 2A, Supplementary Table S1) that: opioidergic genes *Pdyn* was upregulated (*p* = 0.022) and *Pomc* was downregulated (*p* = 0.027); CAergic genes: *Th* (*p* = 0.035) and *Drd2* (*p* = 0.015) were upregulated; GABAergic genes: *Gabrq* was upregulated (*p* = 0.02), and *Slc6a13* was downregulated (*p* < 0.001, *q* = 0.004); Glutamatergic genes: *Grin3a* was downregulated (*p* = 0.045), and *Slc17a7* was upregulated (*p* = 0.007). 

Three gene clusters were identified showing a relationship among DEGs (Figure 2B). The first cluster contains genes whose expression increased (*Slc17a7, Drd2,* and *Pdyn*), and expression of the *Drd2* gene positively correlated with that of genes *Slc17a7* (R = 0.817) and *Pdyn* (R = 0.850) and negatively with the expression of *Grin3a* gene (R = −0.976; Supplementary Table S3). The second cluster comprises genes with decreased expression: *Pomc* and *Slc6a13,* encoding proopiomelanocortin and GABA transporter, respectively. There are no correlations in the expression of these genes with other genes. The third cluster combines genes with increased expression: *Th* and *Gabrq* (R = 0.876), encoding tyrosine hydroxylase and a GABA receptor, *Grin3a,* respectively. The observed strong correlation of gene expression levels indicates transcriptionally coordinated processes.

Similarly, with changes in the VTA, we can assume upregulation of dopaminergic genes *Th* and *Drd2*, of the opioidergic *Pdyn* gene, and of the glutamatergic *Slc17a7* gene in the NAcc. The *Slc17a7* gene codes for a vesicular glutamate transporter (VGLUT1), which mediates the uptake of glutamate into synaptic vesicles at presynaptic nerve terminals of excitatory neural cells and regulates the release of glutamate. 

#### 2.2.3. DEGs in the STR of Highly Aggressive Mice

**Figure 3:**
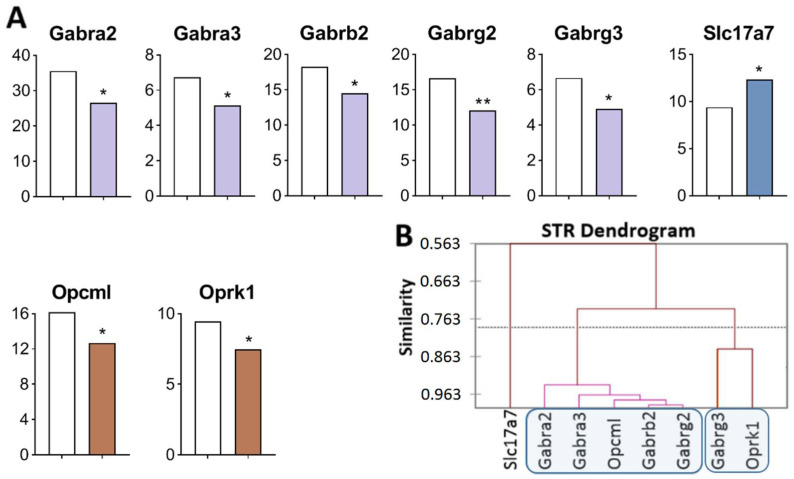
DEGs in the STR of highly aggressive mice. (**A**) FPKM of opioidergic, GABAergic, and glutamatergic DEGs in the STR. White bars—controls; colored bars—winners: brown—opioidergic genes; lilac—GABAergic genes; violet—glutamatergic genes. * *p* < 0.05; ** *p* < 0.01 (Supplementary Table S1). (**B**) AHC based on expression profiles of eight reference DEGs (Supplementary Table S2). Similarity: Pearson’s correlation coefficient. Agglomeration method: Unweighted pair-group average. The main clusters are highlighted with a blue rounded rectangle.

Analysis of the RNA-Seq database of the STR [47] revealed DEGs of interest in this region that participate in rewarding processes (Figure 3A, Supplementary Table S1). Opioidergic genes were downregulated: *Opcml* (*p* = 0.034) and *Oprk1* (*p* = 0.042); GABAergic genes encoding GABA receptors were downregulated: *Gabra2* (*p* = 0.024), *Gabra3* (*p* = 0.029), *Gabrb2* (*p* = 0.042), *Gabrg2* (*p* = 0.005), and *Gabrg3* (*p* = 0.049); Glutamatergic transporter gene *Slc17a7* was upregulated (*p* = 0.047).

Three gene clusters were identified showing a relationship or its absence among DEGs in aggressive mice (Figure 3B; Supplementary Table S2). The first cluster combines genes whose expression declined (*Opcml* encoding an opioid-binding protein, and genes encoding GABA receptors: *Gabra2, Gabra3, Gabrb2,* and *Gabrg2*), and the second cluster is composed of genes *Gabrg3* and *Oprk1*. The third cluster consists of *Slc17a7*. Expression of all genes of interest was found to be downregulated except for the *Slc17a7* gene, a single gene that was upregulated and did not correlate with other genes.

The largest number of correlations was found (Supplementary Table S3) between the *Opcml* gene (encoding an opioid-binding protein) and genes encoding GABA receptors: *Gabra2* (R = 0.972), *Gabra3* (R = 0.938)*; Gabrb2* (R *=* 0.974), *Gabrg2* (R *=* 0.983,), and *Gabrg3* (R = 0.826). The *Gabra2* gene manifested the largest number of correlations. 

#### 2.2.4. DEGs in the PFC of Highly Aggressive Mice 

**Figure 4:**
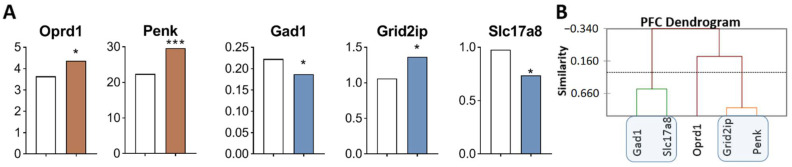
DEGs in the PFC of highly aggressive mice. (**A**) FPKM of opioidergic and glutamatergic DEGs in the PFC of mice. White bars—controls; colored bars—winners: brown—opioidergic genes; violet—glutamatergic genes (Supplementary Table S1). * *p* < 0.05; *** *p* < 0.001. (**B**) AHC based on five reference DEGs’ expression profiles (Supplementary Table S2). Similarity: Pearson’s correlation coefficient. Agglomeration method: Unweighted pair-group average. The main clusters are highlighted with a blue rounded rectangle.

In comparison with the control, opioidergic genes were upregulated (Figure 4A, Supplementary Table S1): *Oprd1* (*p* = 0.028), encoding delta opioid receptors, and *Penk* (*p* < 0.001), encoding pentapeptide opioids Met-enkephalin and Leu-enkephalin. Glutamatergic genes: *Grid2ip* (*p* = 0.041) encoding glutamate receptor was upregulated, whereas *Slc17a8* (*p* = 0.032) and *Gad1* (*p* = 0.044), encoding glutamate transporter and glutamate decarboxylase, respectively, were downregulated.

Three gene clusters were identified showing a relationship or its absence among DEGs in aggressive mice (Figure 4B; Supplementary Table S2). The first cluster combines genes whose expression declined (*Gad1* and *Slc17a8*). The second cluster combines *Grid2ip* and *Penk* genes. The third cluster consists of *Oprd1* gene. Expression of genes of second and third clusters was found to be upregulated.

Correlations of expression levels were found between the *Grid2ip* gene and *Penk* (R = 0.873) and *Slc17a8* (R = −0.934) (Supplementary Table S3).

#### 2.2.5. DEGs in the MRNs of Highly Aggressive Mice 

**Figure 5:**
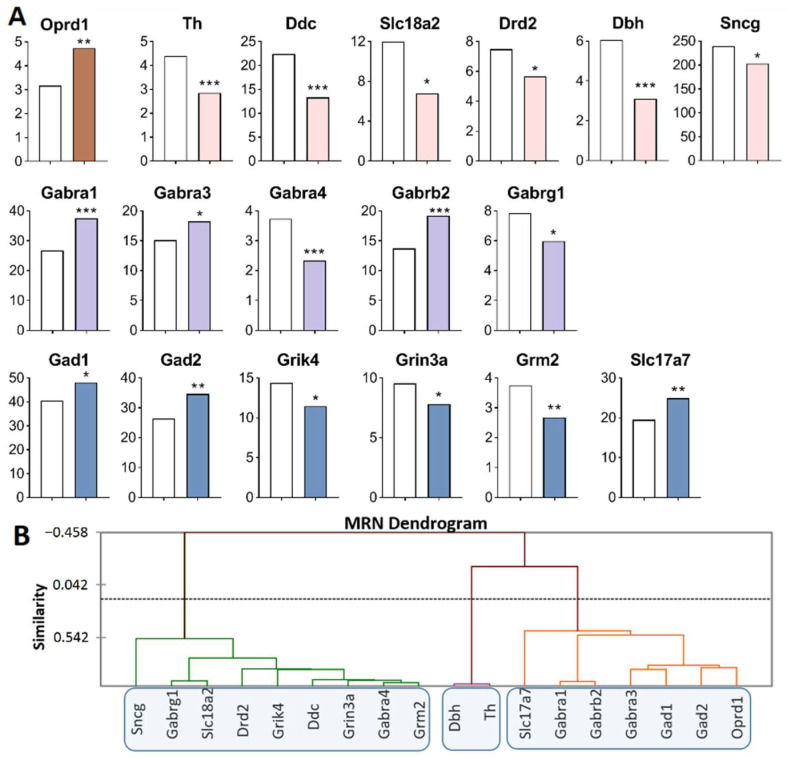
DEGs in the MRNs of highly aggressive mice. (**A**) FPKM of opioidergic, CAergic, GABAergic and glutamatergic DEGs in the MRNs (Supplementary Table S1). White bars—controls; colored bars—winners: brown—opioidergic genes; pink—CAergic genes; lilac—GABAergic genes; violet—glutamatergic genes. * *p* < 0.05; ** *p* < 0.01; *** *p* < 0.001. (**B**) AHC based on expression profiles of 18 reference DEGs (Supplementary Table S2). Similarity: Pearson’s correlation coefficient. Agglomeration method: Unweighted pair-group average. The main clusters are highlighted with a blue rounded rectangle.

Analysis of the RNA-Seq database of MRNs [48] revealed the largest number of DEGs of interest in this main region processes (Figure 5A, Supplementary Table S1). Opioidergic gene *Oprd1* was upregulated (*p* = 0.009) and CAergic genes were downregulated: *Th* (*p* < 0.001; *q* = 0.035), *Ddc* (*p* < 0.001; *q* = 0.005), *Dbh* (*p* < 0.001; *q* = 0.005), *Sncg* (*p* < 0.046), *Drd2* (*p* = 0.03), and *Slc18a2* (*p* = 0.04). GABAergic genes encoding GABA receptors—*Gabra1* (*p* < 0.001; *q* = 0.005), *Gabrb2* (*p* < 0.001; *q* = 0.005), and *Gabra3* (*p* = 0.024)—were upregulated, whereas *Gabrg1* (*p* = 0.043) and *Gabra4* (*p* < 0.001; *q* = 0.005) were downregulated. Glutamatergic genes: *Grm2* (*p* = 0.003), *Grin3a* (*p* = 0.035), and *Grik4* (*p* = 0.011) were downregulated, while Slc17a7 (*p* = 0.004), *Gad1* (*p* < 0.032), and *Gad2* (*p* = 0.001, *q* = 0.05) were upregulated.

Three gene clusters were identified showing a relationship among DEGs in aggressive mice (Figure 5B; Supplementary Table S2). The first cluster combines genes whose expression declined (*Sncg, Drd2, Ddc, Slc18a2, Gabrg1, Gabra4, Grik4, Grin3a* and *Grm2* genes), and the second cluster (*Th* and *Dbh*). The third cluster is composed of upregulated genes *Slc17a7, Gabra1, Gabrb2, Gabra3, Gad1, Gad2*, and *Oprd1*. 

Our finding suggested that the *Slc18a2* gene is associated with a greater risk of opioid dependence [49]. The expression of *Oprd1* positively correlated with that of *Gad2* (R = 0.829) and negatively with the expression of *Grik4* (R = −0.814) in our experiment (Supplementary Table S3). The largest number of correlations with expression levels of various genes in the MRNs belongs to the *Grik4* gene: correlations with *Ddc* (R = 0.862), *Drd2* (R = 0.819), *Gabra1* (R = −0.828), *Gabra3* (R = −0.963), *Gabra4* (R = 0.897), *Gad2* (R = −0.827), *Grin3a* (R = 0.846), and *Oprd1* (R = −0.814). A large number of expression correlations were detected for *Gabra4*: with *Ddc* (R = 0.959), *Drd2* (R = 0.887), *Gabra3* (R = −0.880), *Grik4* (R = 0.897), *Grin3a* (R = 0.966), *Grm2* (R = 0.970), and *Slc18a2* (R = 0.887) (Supplementary Table S3). 

In the MRNs, genes encoding proteins that are involved in neurotransmitter systems—serotonergic [50] and dopaminergic—were downregulated, possibly as consequence of under expression of *Ddc* and *Slc18a2*, coding for the synaptic vesicle monoamine transporter common for the regulation of both systems (Figure 5A).

Table 2 presents the full list of DEGs in brain regions of the mice with positive fighting experience.

### 2.3. PCA Based on CAergic and Opioidergic DEGs’ Expression Profiles in Brain Regions of Highly Aggressive Mice

**Figure 6:**
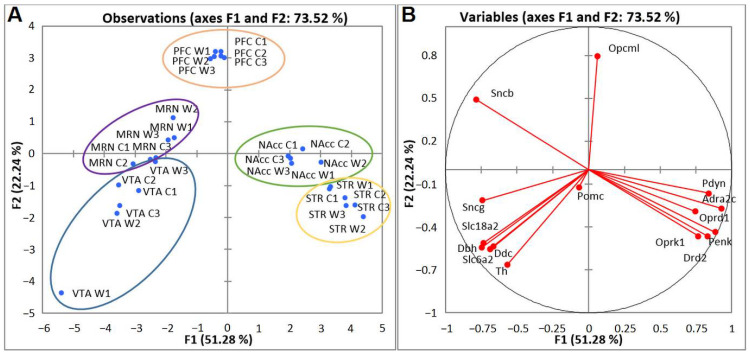
PCA plots based on covariation of genes on the basis of expression profiles of 15 CAergic and opioidergic DEGs across 30 samples, which comprise RNA-Seq FPKM data for five brain regions. (**A**) Active observations. W1, W2, and W3: winners; C1, C2, and C3: controls; VTA: ventral tegmental area, NAcc: nucleus accumbens, MRN: midbrain raphe nuclei, STR: dorsal striatum, and PFC: prefrontal cortex. Ovals denote brain regions. (**B**) Active variables. The graph illustrates distinct clustering of DEGs.

We next analyzed DEGs encoding proteins participating in the functioning of the main neurotransmitter systems of addiction—CAergic and opioidergic—in mice with positive fighting experience. Compact clustering of samples in the NAcc, STR, PFC, VTA, and MRNs on the basis of gene expression profiles can be observed (Figure 6A). Within the circled area, one can see the compact clustering of aggressive vs. control mice; thus, distinct expression patterns of the analyzed genes in each brain region and distinct clustering for the five brain regions were revealed. All genes in the blue cluster are CAergic and have much higher FPKM values in the VTA and MRNs. Most of the genes in the yellow cluster are opioidergic (except *Adra2c* and *Drd2*) and are much more strongly expressed in the STR and NAcc.

PCA biplot analysis based on covariation of the gene expression profiles of six samples for each brain region (Figure 6B) uncovered distinct intergroup clustering of opioidergic and CAergic DEGs. Graphs represent the correlating clusters of DEGs directed oppositely, which means elevated and low levels of gene transcription.

**Figure 7:**
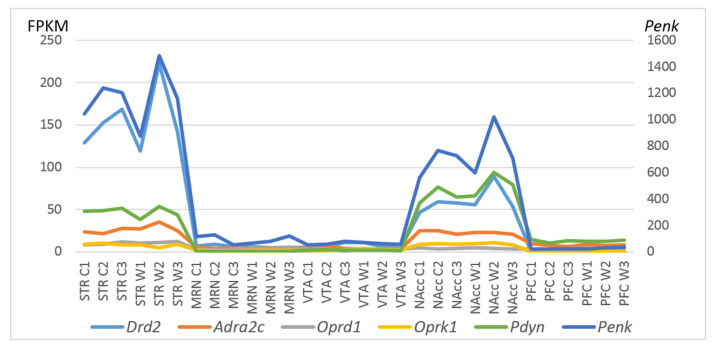
FPKM values of five CAergic- and opioid-specific DEGs across 30 samples on the basis of the included RNA-Seq FPKM data for five brain regions. C1, C2, and C3: controls; W1, W2, and W3: winners; VTA: ventral tegmental area, NAcc: nucleus accumbens, MRN: midbrain raphe nuclei, STR: dorsal striatum, and PFC: prefrontal cortex.

To gain further insight into CAergic–opioidergic interactions, we found the most overexpressed neuron-specific genes in the brain regions under study (Figure 7). Our genes of interest were the CA-specific *Adra2c* and *Drd2* gene and opioid-associated genes *Oprd1, Oprk1, Pdyn,* and *Penk* for comparison, as presented in Figure 7. Both *Drd2* and *Penk* in the STR and *Penk, Pdyn,* and *Drd2* in the NAcc are the most highly expressed DEGs coding neuron-specific genes. These data suggest that all the main correlating events concerning the development of an addiction-like state take place in the STR and NAcc.

## 3. Discussion

It is well known that any positive and long rewarding processes that are accompanied by positive emotions can lead to an addiction-like state, for example, drug abuse, sexual abuse, gaming abuse, etc. Earlier experimental data have led to the conclusion that aggression is rewarding and strongly influenced by experience of aggression: any positive reinforcement in agonistic interactions can cause a propensity to behave aggressively [28,51,52,53,54,55,56,57]. Our data revealed that repeated positive fighting experience in daily agonistic interactions leads to addiction-like signs in mice [29,31] according to criteria proposed for drug dependence and addictive states [58].

In this work and in our previous studies [25,26,45,46,47,48,59], we focus on DEGs encoding proteins that are related to reward-associated neurotransmitters in the brain: opioid-, catecholamine-, GABA-, and glutamatergic systems, which partake in the regulation of aggression in the VTA, STR, and NAcc (which are considered the main regions implicated in aggression stimulation) and in the MRNs and PFC, which as a rule, perform inhibitory functions toward aggressive behavior [6]. 

It has been shown earlier that repeated positive fighting experience is accompanied by overexpression of genes *Th*, *Slc6a3*, and *Snca* in the VTA [25,26]. Our RNA-seq data additionally revealed upregulation of the ***Ddc*** gene, encoding an enzyme of monoamine synthesis, and the ***Slc6a2*** gene, encoding a noradrenaline transporter, and upregulation of glutamatergic genes ***Slc17a7*** and ***Slc17a8***, encoding vesicular glutamate transporters. Correlations were found between the expression of ***Oprk1*** encoding opioid receptor kappa 1 (OPRK1) and ***Ddc*** and ***Slc17a8*** expression levels (Supplementary Table S3). OPRK1’s function is connected with receptors of endogenous ligands, such as α-neoendorphins and dynorphins, as well as receptors of various synthetic opioids. According to the GeneCard database, diseases associated with the OPRK1 protein include morphine and alcohol dependence (https://www.genecards.org/cgi-bin/carddisp.pl?gene=OPRK1 (accessed on 3 July 2021). 

Expression levels of vesicular glutamate transporters SLC17A6 and SLC17A7 are robustly raised by smoking: an effect that is reduced by alcohol coexposure [60]. Those authors proposed that glutamatergic transmission is crucial for the control of the VTA, and that enduring plasticity within the VTA may be a major molecular mechanism for the maintenance, for example, of smoking and alcohol addiction. DA- and cAMP-regulated neuronal phosphoprotein PPP1R1B and vesicular glutamate transporter SLC17A7 may be molecular targets for the treatment of substance abuse [61]. Thus, our findings about upregulation of ***Slc17a7*** in the VTA, NAcc, STR, and MRNs are consistent with the involvement of this DEG in the development of an addiction-like state, in our case, induced by positive fighting experience accompanied by wins in daily agonistic interactions.

In the NAcc of the winners, there were revealed upregulation of dopaminergic genes (***Th*** and ***Drd2***) concurrently with upregulation of glutamatergic gene ***Slc17a7*** and of opioid-related gene ***Pdyn*** (prodynorphin, DYN). Enhanced dopaminergic and glutamatergic signaling in the NAcc are thought to be hallmarks of addiction. Structural and functional alterations in dendritic MSNs within this region and their dopaminergic projections from the VTA are believed to facilitate addiction [62]. 

According to GeneCards data, the protein encoded by ***Pdyn*** gene is a preproprotein that is processed into secreted opioid peptides β-neoendorphin, dynorphin, leu-enkephalin, rimorphin, and leumorphin. These peptides are ligands for the kappa opioid receptor (KOR), and an association has been shown between prodynorphin gene polymorphisms and opioid dependence susceptibility [63]. It has been suggested that dysregulation of the DYN/KOR system and of DA signaling through both alterations in co-expression patterns of opioid genes and decreased *Drd1* mRNA expression can contribute to an imbalance in the activity of D1- and D2-containing pathways thereby possibly causing a negative affective state in human alcoholics [64]. In contrast to these data, in this study, we found upregulation of ***Drd2*** but not *Drd1* mRNA expression in the winners. PDYN binds to κ-opioid receptors encoded by the *Oprk* gene and is known to regulate dopaminergic tone, making this system important for drug addiction. The DYN/KOR system, as supposed by the authors of ref. [65], is a powerful effector of stress-induced alterations in reward processing and dysphoric states. Thus, upregulation of genes ***Th, Drd2, Pdyn****,* and ***Slc17a7*** in the NAcc and of ***Oprk1*** in the VTA of mice with positive fighting experience is consistent with data indicating that this system is implicated in addiction.

NAcc pathways control cell-specific gene expression in the PFC [66], which is highly interconnected with other brain regions, including extensive connections with subcortical and other cortical structures, notably the thalamus, basal ganglia, hypothalamus, amygdala, and hippocampus, among others [67]. Several neurotransmitter systems are represented in the PFC, in particular dopaminergic, glutamatergic, and cholinergic ones [68,69]. Additionally, glutamatergic neurons from the PFC project to MSNs, providing an enriched target for the regulation of synaptic plasticity. 

According to some authors [70,71], the VTA sends glutamate to the PFC. We can suppose the activation of opioid genes ***Oprd1*** and ***Penk*** (proenkephalin), which are paralogs of the ***Pdyn*** gene. The ***Oprd1*** gene encodes opioid receptor delta 1 with functions as a receptor for endogenous enkephalins and for a subset of other opioids. OPRD1 plays a role in the development of analgesic tolerance to morphine. The ***Penk*** gene codes for proteins, including pentapeptide opioids Met-enkephalin and to a lesser extent Leu-enkephalin, that are stored in synaptic vesicles and then released into the synapse, where they bind to mu- and delta-opioid receptors. PENK, according to the GeneCards database (https://www.genecards.org/cgi-bin/carddisp.pl?gene=PENK (accessed on 3 July 2021), participates in morphine, heroin, cannabis, and alcohol dependence. Diseases associated with PENK also include cannabis dependence. We found a significant correlation in expression levels between ***Penk*** and ***Grid2ip*** encoding glutamate receptor genes. There were no GABAergic and CAergic DEGs in the PFC. 

Our earlier RT-PCR study has revealed under expression of the *Th* gene and overexpression of the ***Drd4*** gene in the STR [72]. Moreover, the development of hyperactivity in the winners after long experience of aggression presented in our study may be induced by decreased activity of GABAergic systems, judging by downregulation of *Gabra2, Gabra3, Gabrb2, Gabrg2,* and *Gabrg3* encoding GABA receptors and upregulation of the ***Slc17a7*** gene, implicated in addiction.

The inhibitory influence of the MRNs on aggression supposedly is disturbed during repeated aggression because there is an overall under expression of serotonergic genes -*Tph2* and *Ddc* encoding proteins, participating in the synthesis 5-HT, its transporters (*Slc6a4* and *Slc18a2*), and receptors (*Htr2a, Htr3a,* and *Htr5b*) [50] and of dopaminergic genes—*Th, Comt, Ddc, Dbh, Drd2, Slc18a2*, and *Sncg*—encoding proteins involved in the synthesis and workings of receptors (our current data). This downregulation may be a consequence of inhibition of the neurotransmitter release because of upregulation of the ***Oprd1*** gene, which is associated with glutamatergic transporters, as a result of overexpression of the ***Slc17a7*** gene. 

Our data are consistent with other studies, which indicate that GABAergic and glutamatergic systems are deeply involved in the development of addiction, for example, to amphetamine [73], alcohol, and cocaine [38]. Alcoholics and cocaine addicts show upregulation of three genes relative to controls: *Gria4, Grik3,* and *Grm4*. Expression of both *Grm3* and *Grin2d* is high in alcoholics and low in cocaine addicts. These observations suggest that glutamate input into dorsal raphe nuclei is enhanced during escalated aggression, which causes a phasic increase in the 5-HT release from dorsal raphe nuclei 5-HT neurons into the NAcc and inhibits the reinforcing and motivational effects of cocaine, heroin, and ethanol [4]. 

Thus, our neurotranscriptomic data confirm the development of an addiction-like state during positive fighting experience in the winner. Some KEGG terms turned out to be significantly related to the DEGs associated with an addiction-like state in the VTA, NAcc, STR, MRNs, and PFC of the winners. These terms are nicotine, retrograde endocannabinoid signaling, morphine, cocaine, amphetamine, and alcoholism addiction (Table 3). This study shows that the DEGs that are implicated in the development of opioid dependence and addiction in the winners are *Oprk1* in the VTA (upregulation) and STR (downregulation); *Oprd1* (upregulation) in MRNs and *Oprd1* and *Penk* (upregulation) in the PFC; *Pdyn* gene (upregulation) in the NAcc as well as *Th, Ddc, Slc6a2, Slc6a3,* and *Snca* in the VTA (upregulation) and *Drd2* and *Pdyn* (upregulation) in the NAcc. As markers of changed function of glutamatergic and GABAergic systems in the STR, NAcc, and VTA, we registered overexpression of *Slc17a7* and *Slc17a8* in the VTA and the expression of genes involved in synthesis (except for the STR) of DA. In the MRNs, the expression of dopaminergic and serotonergic genes proved to be reduced or unchanged, but in both regions, the *Oprd1* gene is upregulated, and the PFC is activated by the *Penk* gene. 

Indirectly, all these genes and proteins encoded by them may induce neurotranscriptomic changes in the synaptic vesicle cycle, taste transduction, neuroactive ligand-receptor interaction, tyrosine metabolism, and glutamatergic, GABAergic, and dopaminergic synapses, or vice versa in some cases (Table 3).

Directly, our data prove changes in the expression of opioidergic genes which, according to GeneCards and MalaCards databases (https://www.malacards.org/card/opiate_dependence (accessed on 3 July 2021)), code for proteins implicated in opioid dependence and addiction: that is, OPRM1 (opioid dependence); OPRM1, OPRK1, OPRD1, and DRD2 (opioid addiction); PENK, PDYN, OPRM1, OPRK1, and OPRD1 (morphine dependence); and PDYN, OPRM1, OPRD1, and DRD2 (heroin dependence). The most studied candidate genes have included mu-opioid receptor (OPRM1), delta-opioid receptor (OPRD1), and dopamine D2 receptor (DRD2). Variants in these genes have been associated with relatively small but reproducible effects on addiction risk [74]. All these data support our notion of the development of an addiction-like state as a result of positive fighting experience in daily agonistic interactions in mice. 

The term “addiction” describes a progressive loss of behavioral control leading to dependence on a drug and inability to stop without adverse consequences (American Psychiatric Association, 1994). All abused substances, tend to foster increasing use, threatening to cause dependence. This suggests that all reward stimuli have common properties that both depend on and contribute to altered functioning of the central nervous system. Replacing the word “drugs” with “aggression”, we can say that this quote also applies to the state induced by repeated positive fighting experience. 

## 4. Methods and Materials

### 4.1. Animals

The experiment was carried out using 10–12-week-old C57BL/6J male mice. The mice were kept in the Conventional Vivarium (Federal Research Center Institute of Cytology and Genetics, SB RAS, Novosibirsk, Russia) under standard conditions at 22 ± 2 °C on a 12/12 h light–dark cycle (lights on at 8:00 AM) with dry laboratory feed and water available *ad libitum*. The mice lived in groups of 8–10 in plastic cages (36 × 23 × 12 cm). All procedures were carried out in compliance with the international regulations for animal experiments (Directive 2010/63/EU of the European Parliament and of the Council on the Protection of Animals Used for Scientific Purposes). The protocol for the study was approved by Scientific Council No. 9 of the Institute of Cytology and Genetics, SB RAS, of 24 March 2010, N 613 (Novosibirsk, http://spf.bionet.nsc.ru/ (accessed on 24 March 2010)).

### 4.2. The Sensory Contact Model for the Development of Pathological Aggression in Mice

**Figure 8:**
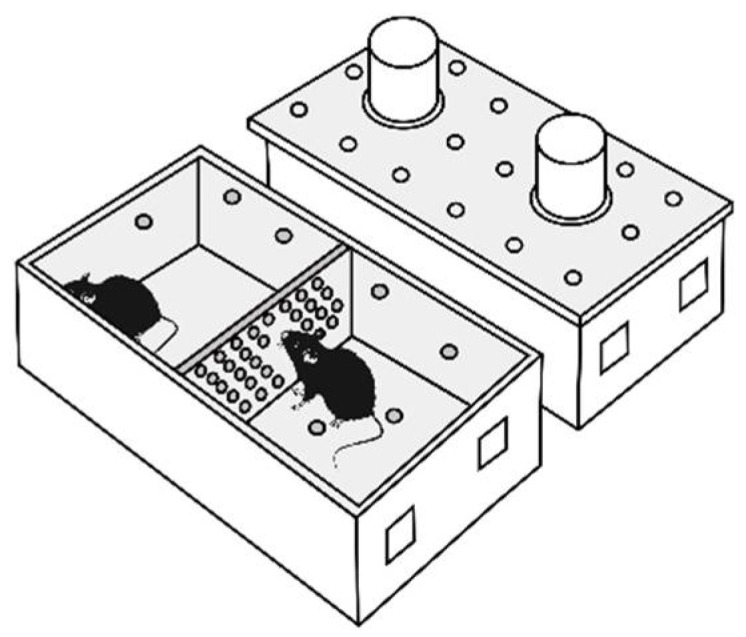
The experimental cage.

Repeated positive fighting experience, i.e., wins, in male mice were induced by daily agonistic interactions [43,44] in chronic social conflicts. Pairs of animals were each placed in a cage (28 × 14 × 10 cm) bisected by a transparent perforated partition allowing the animals to hear, see, and smell each other but preventing physical contact (Figure 8). The animals were left undisturbed for 2 days to adapt to the new housing conditions and sensory contact before they were exposed to agonistic encounters. 

Every afternoon (14:00–17:00 p.m. local time) the cage cover was replaced by a transparent one, and 5 min later (the period necessary for activation), the partition was removed for 10 min to encourage agonistic interactions. The superiority of one of the mice was established within two or three encounters with the same opponent. The superior (winning) mouse would be chasing, biting, and attacking another, who would be demonstrating only defensive behavior (e.g., upright or sideways postures and withdrawal). To prevent physical damage to the defeated mice, the aggressive interactions between males were discontinued by lowering the partition if the strong attacking behavior had lasted for 3 min (in some cases less). Each defeated mouse (loser) was exposed to the same winner for 3 days, whereas afterwards, each loser was placed after the fight in an unfamiliar cage with an unfamiliar winning partner behind the partition. Each aggressive mouse (winners, aggressive mice) remained in its own cage. This procedure was performed once a day for 20 days. 

Two groups of animals were used in this study: (1) controls, i.e., mice without consecutive experiences of agonistic interactions; (2) winners, i.e., chronically aggressive mice. The winners with the most pronounced behavioral aggressive phenotypes were selected for the analysis. They demonstrated the largest number, the longest total attacking time, and hostile behavior during the 20-day experiment (Table 1). At 24 h after the last agonistic interactions, the control animals and the aggressive mice were simultaneously decapitated. Brain regions were dissected by the same experimenter according to the Allen Mouse Brain Atlas map [http://mouse.brain-map.org/static/atlas (accessed on 20 April, 2021)]. All tissue samples were placed in the RNAlater solution (Life Technologies, Waltham, MA, USA) and were stored at −70 °C until sequencing.

### 4.3. Analysis of the Winners’ Behavior during Agonistic Confrontation

Approximately 20–40% of C57BL/6J males displayed strong uncontrolled pathological aggression after the 20-day aggression experience. For analysis, only the winners demonstrating the most pronounced offensive aggression were selected. 

Video recordings were used to describe the behavior of the winners in detail during the agonistic interactions. The Observer XT software (version 7.0; Noldus Information Technology, the Netherlands) was employed for manual registration of the behavioral indicators during the test. The following types of attacking behavior were analyzed: attacks, biting, and chasing a partner in agonistic interactions during 10 min, and the following parameters were measured: (1) latency to the first attack (seconds, s); (2) total time of attacks (s); (3) the number of attacks; (4) average time of one attack (total time/number) (s); (5) digging: here it means digging up and scattering the sawdust on the loser’s territory (kick digging or push digging the sawdust forward or backward by forepaws or hind paws): total digging time (s) and the number of diggings; (6) hostile behavior: the total time of attacking and digging behavior. All these activities were aimed at inflicting physical or psychological damage on the conspecific. Besides, stereotypic behaviors (jerks and unmotivated long self-grooming) were registered as parameters of pathological states. 

### 4.4. High-Throughput RNA Sequencing (RNA-Seq)

The collected brain samples were sequenced at JSC Genoanalytica (www.genoanalytica.ru, Moscow, Russia) (accessed on 21 November 2017), and the mRNA was extracted using the Dynabeads mRNA Purification Kit (Ambion, Thermo Fisher Scientific, Waltham, MA, USA). cDNA libraries were constructed by means of the NEBNext mRNA Library PrepReagent Set for Illumina (New England Biolabs, Ipswich, MA USA) and were subjected to Illumina sequencing. More than 20 million reads were obtained from each sample. The resulting “fastq” format files were used to align all reads to the GRCm38.p3 reference genome in the TopHat aligner [75]. The Cufflinks software was utilized to estimate gene expression levels in FPKM (fragments per kilobase of transcript per million mapped reads) and to subsequently identify differentially expressed genes (DEGs) in the winner group and control group. Each brain area was analyzed in three versus three animals. Genes were considered differentially expressed at *p ≤* 0.05, and these data were corrected for multiple comparisons at *q* < 0.05. 

DAVID Bioinformatics Resources 6.7 (http://david.abcc.ncifcrf.gov (accessed on 20 July 2021) was used to describe DEGs’ functional relations and Gene Ontology terms. The Human Gene Database (http://www.genecards.org/, (accessed on 20 July 2021), Online Mendelian Inheritance in Man database (http://omim.org/, (accessed on 20 July 2021), and a human disease database (MalaCards, http://www.malacards.org, (accessed on 20 July 2021) were employed for the description and analysis of the data obtained. 

### 4.5. Brain Regions That Are Responsible for the Regulation of Aggression and Development of Addiction during Reward

The VTA, which is a critical brain region involved in reward processes and processes of dependence on drugs, such as cocaine, heroin, and ethanol, contains 55–65% of dopaminergic cell bodies [70,76,77,78]. DA is a major neurotransmitter participating in the integration of afferent signals with inhibitory or excitatory inputs, and the VTA gives rise to the dopaminergic mesolimbic and mesocortical pathways that project to the NAcc and PFC, respectively [70,76,79], play an important part in the mediation of rewarding processes and are associated with many types of social behavior. VTA DA-releasing neurons are heterogeneous in their afferent and efferent connectivity and, in some cases, release GABA or glutamate in addition to DA [70,71]. DA is a key neurotransmitter in reward circuitry and reward-guided learning. Physiological activity of GABAergic and cholinergic interneurons is regulated by dopaminergic transmission in a complex manner [80]. It has been suggested that the VTA can act as a hub combining and integrating multimodal signals that contains dopaminergic neurons and transmits excitation to other regions with the help of DA [81], particularly, to the regions we are interested in. 

The NAcc is a principal target of VTA dopaminergic neurons. Most of the neurons in the NAcc are GABAergic medium spiny neurons (MSNs), which express D1-type or D2-type DA receptors [82,83]; ~1–2% are cholinergic interneurons and another 1–2% are GABAergic interneurons. GABA is the predominant neurotransmitter in the NAcc, and GABA receptors are abundant there [84]. GABAergic MSNs play an important role in the processing of reward stimuli [85], and they are regulated by DA from the VTA. The VTA–NAcc circuit is a key detector of rewarding stimuli. This area is considered a critical brain region involved in reward and drug dependence processes [4,86]. Structural and functional alterations in MSNs within the NAcc and its dopaminergic projections from the VTA are believed to facilitate these behavioral sequelae related to neuroadaptations in NAcc MSNs from dopaminergic and glutamatergic pathways in opioid use disorder [62]. NAcc pathways control cell-specific gene expression in the medial PFC [66].

In the STR, MSNs constitute the bulk (95% in mice) of neurons and represent dopaminoceptive GABAergic neurons [87]. Intracellular signal transduction is crucial for the STR participation in motor and behavioral functions as well as all types of addiction. Via correlation analysis, we have succeeded in dissecting Drd1- and Drd2-dopaminoceptive neurons’ gene pathways in winners [47,88] with hyperactive behavior revealed by us before [72] in chronically aggressive mice.

The PFC is highly interconnected with other brain regions including extensive connections with subcortical and other cortical structures, notably the thalamus, basal ganglia, hypothalamus, amygdala, hippocampus, and others [67]. Several neurotransmitter systems are represented in the PFC, in particular dopaminergic, glutamatergic, and cholinergic systems [68,69]. Persistent strengthening of the PFC–NAcc pathway during cocaine-seeking behavior has been documented [86].

The MRNs have a vast impact upon the central nervous system. A large number of neurons in these nuclei are serotonergic [89]. It is reported that glutamate input into dorsal raphe nuclei is enhanced during escalating aggression, thereby causing a phasic increase of the 5-HT release from the 5-HT neurons. It is believed that the MRNs are involved in a variety of reward-related phenomena including drug addiction and mediate primary reinforcement via GABA_A_ receptors [90]. Downregulation of serotonergic genes in the MRNs has been shown in our experiments on winners [50,59].

### 4.6. The Genes That Were Analyzed in Different Brain Regions 

CAergic systems: *Th, Ddc, Dbh, Maoa, Maob, Comt, Slc6a2, Slc6a3, Slc18a2, Snca, Sncb, Sncg, Ppp1r1b, Drd1, Drd2, Drd3, Drd4, Drd5, Adra1a, Adra1b, Adra1d, Adra2a, Adra2b, Adra2c, Adrb1, Adrb2, Adrb3, Adrbk1*, and *Adrbk2*; 

Opioidergic and cannabinoidergic systems: *Pdyn, Penk, Pomc, Pnoc, Oprm1, Oprd1, Oprk1, Opcml, Ogfr, Ogfrl1, Cnr1, Cnr2*, and *Faah*;

GABAergic system: *Gabra1, Gabra2, Gabra3, Gabra4, Gabra5, Gabra6, Gabrb1, Gabrb2, Gabrb3 Gabrg1, Gabrg2, Gabrg3, Gabrd, Gabre, Gabrp, Gabrq, Gabbr1, Gabbr2, Gabrr1, Gabrr2, Gabrr3, Slc6a11*, and *Slc6a13;*

Glutamatergic system: *Gria1, Gria2, Gria3, Gria4; Grik1, Grik2, Grik3, Grik4, Grik5; Grin1, Grin2a, Grin2b, Grin2c, Grin2d, Grin3a, Grin3b, Grm1, Grm2, Grm3, Grm4, Grm5, Grm6, Grm7, Grm8; Grid1 u Grid2; Grid2ip, Gad1, Gad2, Slc17a6, Slc17a7*, and *Slc17a8*;

Expression of serotonergic genes—*Tph2, Ddc, Maoa, Maob, Htr1a, Htr1b, Htr2a, Htr2c, Htr3a, Htr4, Htr5b, Htr6, Htr7, Htr1d, Htr1f, Htr2b, Htr3b u Htr5a, Slc6a4*, and *Slc18a2*—have been previously analyzed by us [70,71].

In Supplementary Table S1, DEGs in FPKM units in all brain regions are presented.

### 4.7. Statistical Analysis

For the transcriptome data, principal component analysis (PCA) was conducted in the XLStat software (www.xlstat.com, accessed on 31 March 2016). PCA was based on the Pearson product moment correlation matrix calculated from FPKM profiles of the analyzed DEGs. We also used Pearson’s correlation as a similarity metric for agglomerative hierarchical clustering (AHC). The agglomeration method was based on an unweighted pair-group average. 

## 5. Conclusions

Previously, it has been suggested [28,29] that accumulation of positive fighting effects day in and day out is accompanied by significant dynamic changes in social behaviors as well as in brain neurotransmitter activity in experienced winners. Repeated manifestation of aggression accompanied by wins and graduate acquisition of winning experience induce multiple long-term changes in neurotransmitter systems of the brain. These alterations arise due to a rearrangement of brain regulatory mechanisms involving (consecutively or simultaneously) neurotransmitters’ synthesis, catabolism, receptors, and now we can say gene activity. We propose that as a result of long positive fighting experience, the balance between activities of the neurotransmitter systems is disturbed as excitation processes beginning to dominate over inhibitory processes in the winners. This imbalance is due to a reduced activity of the serotonergic system and an enhanced activity of dopaminergic systems in the brain. Under these circumstances, a low threshold for aggressive behavior gets established, which is one of the reasons for recurrent aggression (relapse) demonstrated in experiments in male mice [29,31]. Now, we have obtained evidence that numerous genes associated with major neurotransmitter systems in the reward-related brain regions of mice with positive fighting experience are involved in these processes.

## 6. Limitations

The same genes may be upregulated in one brain region and downregulated in others. As an example, the *Oprk1* gene in the VTA (up) and in the STR (down). The *Th* gene was found to be upregulated in the VTA and NAcc and downregulated in the STR and MRNs. This probably means that the expression of genes may depend on a brain region’s function and cell surroundings. This state of affairs creates difficulties with finding the main target for drugs to cure the addictive state. 

Conversely, the same gene may change its expression similarly in several regions: the *Slc17a7* gene proved to be upregulated in the STR, VTA, NAcc, and MRNs, and *Oprd1* is upregulated in the MRNs and PFC. *Sncg* is downregulated in the VTA and MRNs.

It can also be hypothesized that there are dynamic changes of gene expression depending on the duration of agonistic interactions, and these dynamics may be different and specific for every gene, brain region, and time point of analysis after exposure to an experimental factor. At each specific moment, we can look at a certain picture of alterations and relationships of genes, judging by a change in gene expression or its absence. Further studies are necessary to reveal dynamic changes and possible interconnection between pathological aggression and brain expression of opioidergic and other genes associated with neurotransmitter systems in order to understand the mechanisms underlying the role of endogenous opioids in the offense induced by addictive states.

## Figures and Tables

**Table 1:** Agonistic behavior of mice with different levels of aggression.

	Highly Aggressive Mice			Aggressive Mice			
**Attacking behavior**							*p*
Latency, t	17.50	±	4.52	16.18	±	3.25	
Number, n	7.67	±	2.01	7.09	±	0.71	
Total time, t	146.83	±	14.82	65.00	±	7.29	***
Average time, t/n	24.24	±	4.87	9.98	±	1.33	**
Diggings, n	20.33	±	1.26	24.46	±	1.45	*
Diggings, t	64.17	±	6.97	78.82	±	7.71	
**Hostile behavior**	213.00	±	10.41	155.36	±	8.43	**
Number of mice		6			11		

* *p* < 0.05, ** *p* < 0.01, *** *p* < 0.001 vs. highly aggressive mice.

**Table 2:** DEGs in brain regions of the mice with positive fighting experience.

VTA ^#^	
Opioidergic system	***Oprk1*** *Pomc*
CAergic systems	***Th^+^ Ddc Slc6a2 Slc6a3 ^+^ Snca ^+^*** *Sncb Sncg Adra2c*
Glutamatergic system	*Grid2ip Grm4 **Slc17a7 Slc17a8***
GABAergic system	*Gabra1 Gabrg2*
Serotonergic system *	***Tph2 Ddc Slc6a4***
NAcc	
Opioidergic system	*Pomc, **Pdyn***
Dopaminergic systems	***Th Drd2***
Glutamatergic system	*Grin3a **Slc17a7***
GABAergic system	***Gabrq*** *Slc6a13*
Serotonergic system *	*Htr2a Htr4*
STR **^#^**	
Opioidergic system	*Opcml Oprk1*
Dopaminergic systems	*Th^+^ **Drd4 ^+^***
Glutamatergic system	***Slc17a7***
GABAergic system	*Gabra2 Gabra3 Gabrb2 Gabrg2 Gabrg3*
Serotonergic system *	**-**
PFC	
Opioidergic system	***Oprd1 Penk***
Dopaminergic systems	***-***
Glutamatergic system	***Grid2ip*** *Gad1 Slc17a8*
GABAergic system	**-**
Serotonergic system *	**-**
MRNs ^#^	
Opioidergic system	***Oprd1***
CAergic systems	*Th Comt^+^ Ddc Dbh Drd2 Slc18a2 Sncg*
Glutamatergic system	*Grin3a Grik4 Grm2 **Gad1 Gad2 Slc17a7***
GABAergic system	***Gabra1 Gabra3*** *Gabra4 **Gabrb2** Gabrg1 **Gabrg2***
Serotonergic system *	*Tph2 Ddc Slc6a4 Slc18a2 Htr2a Htr3a Htr5b*

**Bold font**: upregulated genes; regular font: downregulated genes. * These data are presented in ref. [50]. **^+^** These data were obtained by the RT-PCR method [26,27]; ^#^ full transcriptomic data are presented in [45,46,47,48].

**Table 3:** KEGG terms related to the DEGs in the VTA, NAcc, STR, MRNs, and PFC.

KEGG Term	FDR	No.	Gene List
Nicotine addiction	1.44 × 10^−18^	12	*Gabra1, Gabra2, Gabra3, Gabra4, Gabrb2, Gabrg1, Gabrg2, Gabrg3, Gabrq, Slc17a7, Slc17a8, Grin3a*
Retrograde endocannabinoid signaling	1.21 × 10^−10^	11	*Gabra1, Gabra2, Gabra3, Gabra4, Gabrb2, Gabrg1, Gabrg2, Gabrg3, Gabrq, Slc17a7, Slc17a8*
Morphine addiction	1.71 × 10^−9^	9	*Gabra1, Gabra2, Gabra3, Gabra4, Gabrb2, Gabrg1, Gabrg2, Gabrg3, Gabrq*
Cocaine addiction	3.54 × 10^−8^	7	*Th, Ddc, Drd2, Pdyn, Grm2, Grin3a, Slc18a2*
Amphetamine addiction	2.38 × 10^−4^	5	*Th, Ddc, Pdyn, Slc18a2, Grin3a*
Alcoholism	0.001	6	*Th, Ddc, Drd2, Pdyn, Slc18a2, Grin3a*
Synaptic vesicle cycle	3.25 × 10^−4^	5	*Slc6a2, Slc6a13, Slc17a7, Slc17a8, Slc18a2*
Taste transduction	5.85 × 10^−4^	5	*Gabra1, Gabra2, Gabra3, Gabra4, Grm4*
Glutamatergic synapse	1.12 × 10^−4^	6	*Slc17a7, Slc17a8, Grm2, Grm4, Grin3a, Grik4*
GABAergic synapse	1.37 × 10^−14^	12	*Gabra1, Gabra2, Gabra3, Gabra4, Gabrb2, Gabrg1, Gabrg2, Gabrg3, Gabrq, Slc6a13, Gad1, Gad2*
Neuroactive ligand-receptor interaction	1.44 × 10^−18^	20	*Drd2, Oprd1, Oprk1, Penk, Pdyn, Pomc, Adra2c, Gabra1, Gabra2, Gabra3, Gabra4, Gabrb2, Gabrg1, Gabrg2, Gabrg3, Gabrq, Grm2, Grm4, Grik4, Grin3a*
Tyrosine metabolism	0.018	3	*Th, Ddc, Dbh*
Dopaminergic synapse	0.020	4	*Th, Ddc, Drd2, Slc18a2*

Blue color: genes that are directly involved in drug addiction processes. FDR: false discovery rate.

## Data Availability

The additional statistics on the obtained data used to support the findings of this study are available in Supplementary Table S1 (differentially expressed genes in FPKM units) and are cited at relevant places within the text. The other datasets generated during the current study are available from the corresponding author on reasonable request.

## Supplementary Materials

The following supporting information can be downloaded at: https://www.mdpi.com/article/10.3390/ijms232113644/s1.

## Abbreviations

5-HT	serotonin
AHC	agglomerative hierarchical clustering
C	control
W	winners
CAergic	catecholaminergic
FPKM	fragments per kilobase of transcript per million mapped reads
GABA	γ-aminobutyric acid
MRNs	midbrain raphe nuclei
MSNs	medium spiny neurons
NAcc	nucleus accumbens
PCA	principal component analysis
PFC	prefrontal cortex
STR	dorsal striatum
VTA	ventral tegmental area
